# Amyloid Associated Intermittent Network Disruptions in Cognitively Intact Older Subjects: Structural Connectivity Matters

**DOI:** 10.3389/fnagi.2017.00418

**Published:** 2017-12-19

**Authors:** Susanne G. Mueller, Michael W. Weiner

**Affiliations:** ^1^Center for Imaging of Neurodegenerative Diseases, San Francisco Veterans Affairs Medical Center, San Francisco, CA, United States; ^2^Department of Radiology and Biomedical Imaging, University of California, San Francisco, San Francisco, CA, United States

**Keywords:** amyloid, intermittent, functional connectivity, cognitively intact, hypersynchrony, resting state fMRI, DTI, gray matter map

## Abstract

Observations in animal models suggest that amyloid can cause network hypersynchrony in the early preclinical phase of Alzheimer's disease (AD). The aim of this study was (a) to obtain evidence of paroxysmal hypersynchrony in cognitively intact subjects (CN) with increased brain amyloid load from task-free fMRI exams using a dynamic analysis approach, (b) to investigate if and how hypersynchrony interferes with memory performance, and (c) to describe its relationship with gray and white matter connectivity. Florbetapir-F18 PET and task-free 3T functional and structural MRI were acquired in 47 CN (age = 70.6 ± 6.6), 17 were amyloid pos (florbetapir SUVR >1.11). A parcellation scheme encompassing 382 regions of interest was used to extract regional gray matter volumes, FA-weighted fiber tracts and regional BOLD signals. Graph analysis was used to characterize the gray matter atrophy profile and the white matter connectivity of each subject. The fMRI data was processed using a combination of sliding windows, graph and hierarchical cluster analysis. Each activity cluster was characterized by identifying strength dispersion (difference between pos and neg strength) their maximal and minimal pos and neg strength rois and by investigating their distribution and association with memory performance and gray and white matter connectivity using spearman rank correlations (FDR *p* < 0.05). The cluster analysis identified eight different activity clusters. Cluster 8 was characterized by the largest strength dispersion indicating hypersynchrony. Its duration/subject was positively correlated with amyloid load (*r* = 0.42, *p* = 0.03) and negatively with memory performance (CVLT delayed recall *r* = −0.39 *p* = 0.04). The assessment of the regional strength distribution indicated a functional disconnection between mesial temporal structures and the rest of the brain. White matter connectivity was increased in left lateral and mesial temporal lobe and was positively correlated with strength dispersion in the cross-modality analysis suggesting that it enables widespread hypersynchrony. In contrast, precuneus, gray matter connectivity was decreased in the right fusiform gyrus and negatively correlated with high degrees of strength dispersion suggesting that progressing gray matter atrophy could prevent the generation of paroxysmal hypersynchrony in later stages of the disease.

## Introduction

Abnormal functional connectivity measured by task-free fMRI is one of the earliest manifestations of amyloid positivity in cognitively intact subjects (e.g., Sperling et al., 2009; Sheline et al., 2010; Jack et al., 2013; Wang et al., 2013; Brier et al., 2014; Steininger et al., 2014; Jones et al., 2016). Findings in animal models suggest that the early preclinical phase of Alzheimer's disease (AD) before the onset of cognitive impairment is characterized by hypersynchrony, i.e., an increased tendency for synchronous firing of larger than normal neuron populations that has been linked to increased connectivity or hyperconnectivity in task-free fMRI (Palop and Mucke, 2016; Shah et al., 2016). The findings in human studies in this early preclinical phase are far from consistent with some studies findings increased connectivity (e.g., Lim et al., 2014; Matura et al., 2014; Jiang et al., 2016; Schultz et al., 2017; Sepulcre et al., 2017) and others decreased connectivity (e.g., Sheline et al., 2010; Wang et al., 2013; Steininger et al., 2014; Elman et al., 2016) or both but in different regions (Mormino et al., 2011). Differences between study populations, techniques used to assess functional connectivity, regions investigated etc., undoubtedly contribute to these conflicting findings. However, it is also possible that these discrepancies reflect the true nature of abnormal functional connectivity at this early stage. In all these studies it is usually assumed that hyperconnectivity or hypoconnectivity are sustained. An alternative explanation is that hyper- and/or hypoconnectivity are paroxysmal and therefore that the observation of one or the other just reflects the preferred state of the brain at the time of the exam. There is evidence that this is indeed the case for the early stage hyperconnectivity. For example it has been shown in animal models of AD and also in patients suffering from familial AD that high levels of amyloid and amyloid precursor protein can cause intermittent neuronal hyperactivity in form of epileptic discharges (Palop et al., 2007; Palop and Mucke, 2010; Busche et al., 2012; Grienberger et al., 2012; Mucke and Selkoe, 2012; Talantova et al., 2013; Vossel et al., 2013; Kellner et al., 2014; Born, 2015; Stargardt et al., 2015). Only about 2% of the patients diagnosed with mild cognitive impairment (MCI) or dementia due to sporadic AD show overt epileptiform discharges during routine EEG recordings though. Intermittent unspecific EEG abnormalities, e.g., episodic focal or diffuse slowing of the background activity, indicative for low level focal or diffuse paroxysmal hypersynchrony are more common in MCI and AD and can be found in 20–45% of the routine EEGs (Liedorp et al., 2009, 2010; Kramberger et al., 2013). If paroxysmal hypersynchrony severe enough to be detected by routine EEG occurs in the more advanced stages, it is very well-possible that it is already present in a more subtle form in the preclinical stage and contributes to the conflicting resting state findings depending on its severity and frequency in the study population. Furthermore, complex interictal epileptiform discharges as well as diffuse unspecific EEG abnormalities have been associated with impaired cognitive performance (Smits et al., 2011; Kleen et al., 2013) which raises the possibility that also more subtle types of hypersynchrony in the preclinical stages of AD could already have a negative impact on cognition.

The traditional type of task-free fMRI analysis calculates functional connectivity from the correlation of the BOLD signal fluctuations across the whole acquisition time and thus makes the implicit assumption that these fluctuations stay stable during this time. In diseases that are known or suspected to be associated with paroxysmal events short, infrequent hyperconnectivity phases are likely to be canceled out by longer phases of physiological activity or cause unspecific connectivity disturbances. Studies interested in investigating the dynamic behavior of the BOLD signal therefore use modifications of the traditional stationary approach of which the sliding window approach is one of the most commonly used (Hutchison et al., 2013).

The overall objective of this study was to use a combination of sliding windows with graph and cluster analysis to seek evidence for paroxsymal focal or diffuse hyperconnectivity suggesting paroxysmal low level hypersynchrony in cognitively normal elderly subjects with and without increased brain amyloid load and to characterize its spatial pattern. The second objective was to investigate if these phases could affect cognition by correlating their duration with memory performance and by investigating to what degree lateral and mesial temporal lobe structures, i.e., structures not only known to be involved in memory processes but also to be affected early in AD, participate in these phases. It was assumed that hyperconnectivity phases are more likely to have a negative impact on memory performance if they last longer and if this activity interferes with mesial temporal lobe connectivity. The third and last objective was to investigate a potential relationship between structural (gray and white matter) connectivity and the severity of the functional hyperconnectivity phases. It was assumed that structural connectivity had to be intact to enable network hypersynchrony, i.e., that gray matter atrophy due to synapse and/or neuron loss would prevent the brain from generating the type of abnormal firing associated with hypersynchrony and that white matter damage would prevent the synchronization of remotely abnormally firing regions.

## Methods

### Study population

A total of 47 elderly (60 years and older), cognitively intact subjects who were recruited from local Memory Clinics and the community with flyers and advertisements in local newspapers participated in this study. Exclusion criteria included any poorly controlled medical illness (untreated diabetes, hypertension, thyroid disease) and/or use of medication or recreational drugs that could affect brain function, a history of brain trauma, brain surgery or evidence for ischemic events (stroke but not white matter hyperintensities or small lacunes) and skull defects on the MRI. Normal cognitive functioning was assessed by a battery of standard tests that included the Mini Mental State Examination (MMSE), Clinical Dementia Rating (CDR), California Verbal Learning Test II (CVLT-II), the Wechsler Adult Intelligence Scale III (WAIS III, digit symbol, matrix reasoning), and the Delis-Kaplan Executive Function System (DKEF, trail making, verbal fluency, design fluency). From this battery, three CVLT-II subtests Short Free Discriminability and Delayed Recall Discriminability, were chosen to assess the impact of amyloid-associated hyperactivity on cognitive function because an association between these measures and brain structure had been demonstrated in a previous study in this cohort (Mueller et al., 2011). Please see Table 1 for demographic details of the final study population. All participants underwent structural and functional MR imaging and had a florbetapir exam to determine the amyloid beta plaque load. The study was approved by the committees of human research at the University of California, San Francisco (UCSF) and VA Medical Center San Francisco, and written informed consent was obtained from all subjects according to the Declaration of Helsinki.

**Table 1:** Subject characteristics.

	**Amyloid negative**	**Amyloid positive**
	***n* = 30**	***n* = 17**
Age	70.2 (6.3)	72.1 (6.3)
SUVR	1.02 (0.06)	1.25 (0.11)*
ApoE4 pos/neg	7/23	8/11
CDR	0.0 (0.00)	0.00 (0.0)
MMSE	29.8 (0.5)	29.6 (0.8)
CVLT-II immediate recall discriminability	2.3 (0.4)	2.4 (0.7)
CVLT-II short free recall discriminability	2.6 (0.7)	2.5 (0.9)
CVLT-II delayed recall discriminability	2.6 (0.7)	2.5 (0.9)
DKEF verbal fluency	13.9 (3.33)	13.9 (3.4)
Digit symbol	65.6 (17.1)	64.1 (12.2)

### PET

Florbetapir F18 PET exams were acquired either at the VA Medical Center, San Francisco on a GE Discovery 690 PET scanner or at the China Basin Campus of the University of California, San Francisco on a GE Discovery STE VCT PET system. The participants were injected with 10 mCi (370 MBq) of florbetapir followed by 10 min PET acquisition 50 min later. The images were reconstructed and normalized to a florbetapir template on which several gray matter regions of interest known to be vulnerable to amyloid deposition in the temporal and parietal lobes, precuneus and anterior and posterior cingulate were labeled. The mean count from each of these regions was extracted and regional Standard Uptake Value Ratios (SUVR) were calculated using whole cerebellum as the reference region. The global SUVR was calculated by averaging the SUVRs from all cortical labels. Participants with a global SUVR equal or higher than 1.10 were considered to be amyloid positive.

### MR acquisition

All images were acquired on a Siemens Skyra 3T MR system equipped with a 20 channel receive coil. The following sequences were obtained as part of a larger research protocol. (1) T1-weighted gradient echo MRI (MPRAGE) of entire brain, TR/TE/TI = 2300/2.96/1,000 ms, 1.0 × 1.0 × 1.0 mm^3^ resolution, acquisition time = 5.30 min for tissue segmentation. (2) PD/T2 weighted 2D turbo spin-echo sequence, TR = 3,210, TE1/2 = 101/11 ms, 1.0 × 1.0 × 3.0 mm^3^ resolution, acquisition time: 3.43 min, for co-registration between T1 and EPI data. (3) 2D gradient echo EPI sequence TR/TE = 3,000/30 ms, flip angle = 80, 2.5 × 2.5 × 3 mm resolution, no gaps, acquisition time = 8.00 min for dynamic task-free analysis. Subjects were instructed to refrain from caffeinated beverages on the day of the exam, to close their eyes and to relax but stay awake and think of nothing in particular during the scan. (4) EPI-based diffusion weighted imaging (TR/TE, = 7,200/73 ms, 2 × 2 × 2 resolution, 64 diffusion encoding directions with b = 1,000 s/mm^2^, acquisition time: 7.2 min.

### Image processing

#### Task free functional imaging data

The first six time frames were discarded to allow the MRI signal to achieve T1 equilibrium. The remaining 154 timeframes/subject underwent slice time correction, motion correction and realignment onto a mean EPI image in the T1 space, spatial normalization using the transformation matrices generated during the warping of the gray matter maps onto the gray matter template with re-sampling to a 1.5 × 1.5 × 1.5 mm resolution. Framewise displacement (Power et al., 2012) was used to assess the motion during the exam. Conn 17a (www.nitrc.org/projects/conn, Whitfield-Gabrieli and Nieto-Castagnon, 2012) a SPM based toolbox for task and task-free fMRI analysis was used for further processing including linear detrending and band pass filtering (0.008–0.09 Hz) with simultaneous denoising. The latter included the aCompCorr routine to reduce the effects of physiological noise (eroded white and csf maps, five components each) and motion regression (six affine motion parameters and six first order temporal derivatives). In addition to that, ART as implemented in the conn preprocessing was used to identify timeframes with motion exceeding a movement threshold of 0.9 mm which ensures that conn disregards these timeframes during the denoising procedure but leaves the original time series intact. No global signal removal was performed since this is known to falsely increase anti-correlations between time series (Murphy et al., 2009). The AICHA atlas was used to extract the denoised mean time series and to estimate the functional connectivity.

#### Stationary or time average analysis

Timeframes identified as having excessive motion by ART were removed and the functional connectivity matrix calculated. The routines provided by the Brain Connectivity Toolbox (https://sites.google.com/site/bctnet), in particular the weight conserving measures “strength,” was used for this purpose (Rubinov and Sporns, 2011). Weight conserving measures have the advantage that they can be applied to fully connected networks, i.e., it is not necessary to define an arbitrary threshold to generate the type of sparse network required by the more commonly used non-weighted equivalents degree. Strength is defined as the sum of weights of links connected to a node or roi. A roi has a high pos strength if its BOLD fluctuations are positively correlated with a large number of that of other rois and a high negative strength if its BOLD fluctuations are negatively correlated with a large number of that of other rois. Positive (Spos) and negative (Sneg) nodal strength and nodal strength dispersion (Sdisp), defined as difference between nodal positive and negative strength, were used to assess the effects of group, age, and SUVR on functional connectivity. Sdisp increases when Spos increases and Sneg simultaneously decreases, i.e., shows a strength profile consistent with hyperconnectivity or hypersynchrony, and was therefore used as a proxy of hypersynchrony.

#### Dynamic analysis

**Figure 1:**
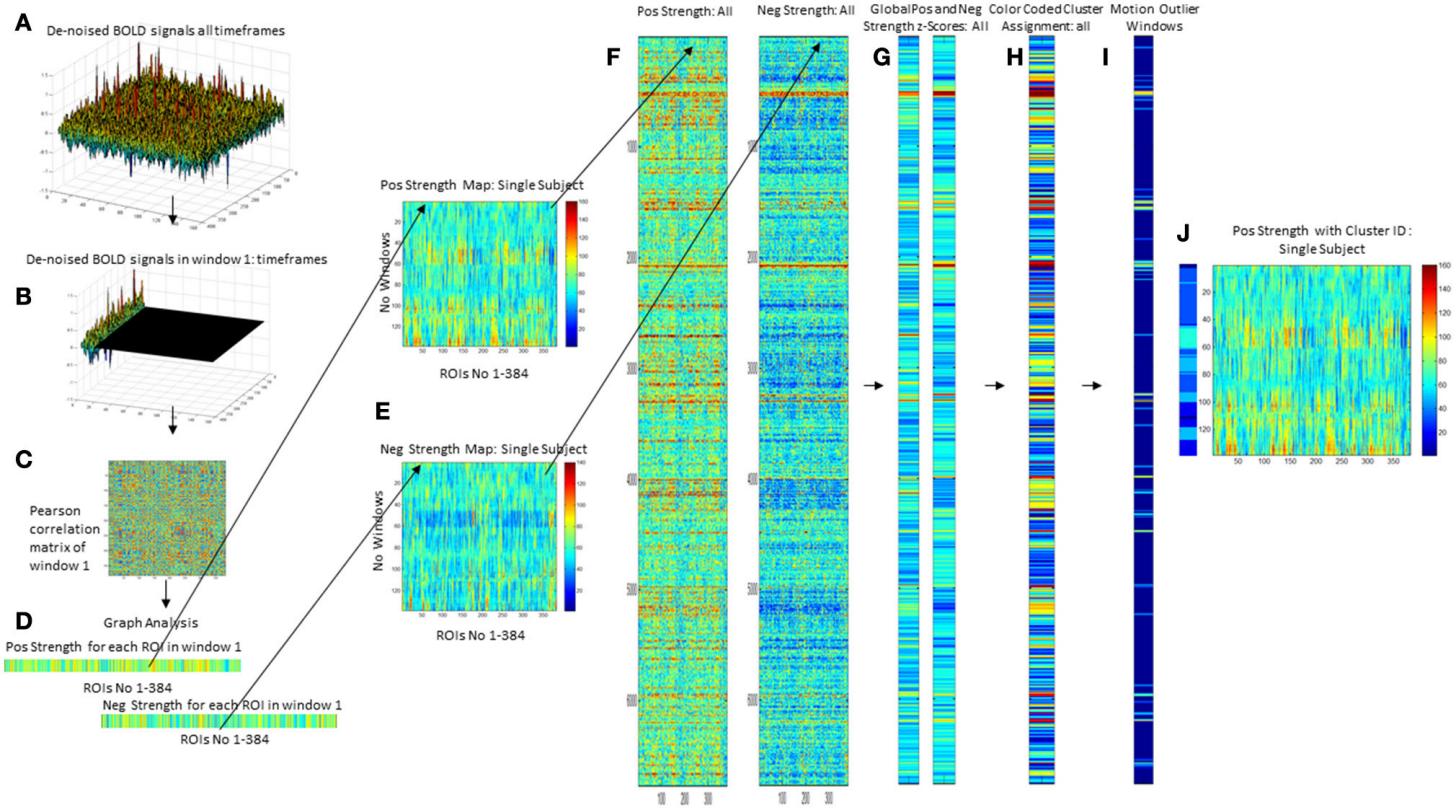
Overview of processing steps. An example of denoised BOLD signal timeseries of an amyloid neg subject is depicted on the upper right side **(A)**. The timeseries is divided into overlapping 45 s long epochs or windows using a sliding windows approach **(B)**, and a correlation matrix calculated using Pearson correlation **(C)**. Graph analysis is used to describe the interactions between the different ROIs for each window **(D)** that are combined to obtain maps that depict the fluctuations of positive (pos) and negative (neg) strength over the subject's whole timeseries **(E)**. For the analysis the strength maps of all 47 subjects were combined **(F)**, converted into z-scores using the mean and std from the amyloid neg CN group as a reference from which the mean from all nodes is calculated for pos strength and neg strength **(G)**. This is used as the input for a hierarchical cluster analysis to identify windows with a similar global pos and neg strength profile **(H)**. The output from ART is used to identify windows with excessive motion or global signal fluctuations **(I)** and to eliminate them from all further analyses. The right-most panel shows the cluster assignment for the sample subject **(J)**. Each of the 138 windows in this subjects strength maps has been assigned to a cluster which allows to determine how many different clusters or activity states occur in each subject and how long they last (duration = no of windows assigned to the cluster/activity state).

A sliding windows approach was used to explore temporal variations of functional connectivity. Based on observations that robust estimations of the functional connectivity without loss of potentially interesting fluctuations are possible with window sizes around 30–60 s (Hutchison et al., 2013), a window with the size of 45 s (15 timeframes) that was advanced with increments of one TR along the artifact corrected time series was chosen resulting in 138 windows/subject or 6,486 windows for all 47 subjects that were converted into 6,486 correlation matrices using Pearson correlation (cf Figures 1A–C).

Graph analysis was again used to describe the interactions between the different nodes in each window (please see Figure 1D). The strength outputs for each window were combined to obtain a map showing the fluctuations of pos and neg strength over the whole acquisition time for each roi for each subject (Figure 1E) and then concatenated across subjects (Figure 1F) to obtain population maps of pos and neg strength (Figure 1F). The nodal positive and negative strength in each window of this population map were converted into z-scores using mean and standard deviation of the nodal strength of the amyloid neg CI as reference with the following formula: strength z-score of node x in window *n* = strength of node x in window *n* – mean of strength of node x from all windows in reference data set/standard deviation of strength of node x from all windows in reference data set. The thus calculated nodal z-scores/window were averaged over all nodes to obtain global positive and negative strength z-scores for each window in each subject (Figure 1G). Hierarchical cluster analysis (Ward's minimum variance methods with the cubic clustering criterion to identify optimal cluster number) was used to identify different global negative and positive strength profiles in amyloid pos and neg subjects (optimal cluster number = 11, please see Figure 1H. The output generated by ART was used to identify windows with motion outliers in the real data and to calculate the percentage of motion outliers for each cluster. Clusters 9–11 consisted of more than 50% of motion outliers (range 75–100%) and therefore were considered to represent “motion clusters” and were not further evaluated. This also eliminated windows that despite not meeting the ART threshold for motion outliers themselves had a similar graph analytical profile as windows that did meet that threshold and were therefore likely to be affected by subthreshold motion. Forty-one percent of the windows assigned to cluster 1 had been identified as motion outliers and therefore cluster 1 was considered as “motion contaminated.” All other clusters had 20% or less motion outliers and were together with cluster 1 fully evaluated after excluding all motion outlier windows. Eliminating windows with excessive motion results in a more rigorous elimination of motion artifacts than just eliminating the motion affected timeframe alone because it also eliminates timeframes with subthreshold motion that usually accompany timeframes with suprathreshold motion (Figures 1I,J).

The last step was to investigate if certain clusters tended to occur together. This was done by calculating the “cluster neighborhood” or the frequency by which a window that had been assigned to cluster A were next to a window assigned to one of the other clusters in a data set. The frequencies with which the other clusters appeared were compared with Fisher's exact test (*p* < 0.05 with Bonferroni correction for multiple comparisons) to identify those cluster(s) that were significantly more often found in the neighborhood of cluster A than others clusters. Table 2 summarizes the global strength profiles of the eight clusters expressed as z-scores. Clusters 1–8 were further characterized by investigating the following features.

1. At the global level by investigating the relationship between SUVR and memory performance (short and delayed recall of the CVLT) and the time (counts of windows assigned to cluster/subject) each strength profile or activity type could be observed in an individual CN.
2. At the nodal level by determining mean nodal strength dispersion for each cluster to investigate differences between the distribution of brain regions with high and low strength dispersion between clusters. Given the a priori hypotheses re memory, the distribution within the medial temporal regions (hippocampus, parahippocampus and fusiform gyrus) and lateral temporal regions (superior, middle and inferior temporal gyrus) was of particular interest. Finally, the relationship between the time a particular strength dispersion profile was observed in an individual CN and SUVR and memory performance was investigated.

**Table 2:** Global strength profiles of all non-motion clusters.

**Cluster ID**	**NoWindows**	**meanSposZ**	**meanSnegZ**	**Median fwd**	**Neighbors**	**No amyloid neg vs. pos**	**Strength profile type**
1	163	−0.116	0.438	0.258	**2**, 3	19/9	Non-balanced negative
2	909	−0.097	0.086	0.241	**4**	28/17	Non-balanced negative
3	926	−0.490	0.155	0.192	**4**, 2	23/16	Non-balanced negative
4	1,202	−0.332	−0.069	0.224	**5**, 2, 3	30/16	Balanced
5	928	−0.182	−0.259	0.232	**4**, 7	28/17	Balanced
6	452	0.362	−0.197	0.236	**7**, 2	23/12	Non-balanced negative
7	775	0.102	−0.476	0.236	**5**, 8, 6	22/14	Non-balanced negative
8	302	0.618	−0.727	0.253	**7**, 6	14/7	Non-balanced negative

*fwd, framewise displacement. please see text for details. Neighboring clusters, bold, cluster that appears most often together with this cluster, non-bold clusters also appearing together with this cluster but less often than bold cluster*.

#### Volumetric imaging data

The T1 images were segmented using the new segmentation algorithm as implemented in SPM12. The gray matter maps were warped onto a symmetrical gray matter atlas in MNI space while preserving the total amount (modulation) using SPM12's DARTEL routine and corrected for intracranial volumes (ICV) using the individual's combined gray/white/csf volumes. The resulting gray matter maps were then converted into z-score maps using the mean and standard deviation of the gray matter maps of 32 healthy young controls (mean age: 28.2 ± 6.7) who had been studied with the same sequence on the same magnet and whose images had undergone the same processing. The atlas of intrinsic connectivity of homotopic areas (AICHA, Joliot et al., 2015) consisting of 384 homotopic cortical, and subcortical gray matter regions of interest (gm roi) was used to extract the mean z-score values for each gm roi. In order to investigate how the mean gray matter z-score in one region is related to that of other regions the so-called profile similarity index (PSI) was calculated. The PSI between gm roi x and gm roi y was defined as follows:

$$\begin{array}{l} \text{rawPSI}=({\text{cROIA-mean}}_{\text{roi}})/\text{abs}(({\text{gm roi x-mean}}_{\text{roi}})\\ \;-({\text{gm roi y-mean}}_{\text{roi}})) \end{array}$$

cROIA is either gm roi x or gm roi y whichever is larger, mean_roi_, is mean over all 384 gm rois.

The rawPSI is calculated for each and every combination between two gm rois resulting in a 384 × 384 matrix for every subject. RawPSI-values exceeding the 95 percentile of all PSI-values in the map are replaced by the PSI-value at the 95 percentile to remove outliers caused by a difference of 0 or very small differences between two gm rois. The rawPSI map is then converted into the final PSI map by multiplying it with a normalization term *n* defined as *n* = 1/(range of all raw PSI in map). A negative PSI indicates a gray matter loss and a positive PSI a gray matter increase relative to the subject's mean z-score. The resulting PSI map of a healthy older individual is determined by atrophic changes due to normal aging. Although an individual PSI map is defined by individual anatomical features, it is assumed to share many features with PSI maps of other healthy elderly subjects. A pathological process though, e.g., gray matter loss in medial temporal structures due to tau pathology, will change the appearance of the PSI map. Graph theory is used to characterize each subject's PSI map. A gm roi has a high strength if it has experienced a similar degree of gray matter loss as the majority of the other gm rois and a low strength if there are only few other gm rois with similar gray matter atrophy. This project focused on differences of negative strength (sSneg) since the focus of this study was on additional gray matter atrophy due to a pathological process. The influence of group (amyloid pos CN vs. amyloid neg CN), age and SUVR on nodal negative gray matter strength was investigated.

#### Diffusion weighted imaging data

ExploreDTI (Leemans et al., 2009) was used to process the DTI data. After correction for motion and eddy-current induced geometric distortions (Leemans and Jones, 2009; Irfanoglu et al., 2012), diffusion tensors were calculated using a non-linear regression procedure. Whole brain fiber tracking was done using a deterministic streamline method and fiber pathways reconstructed by defining seed points uniformly throughout the brain (FA thresholds = 0.2, angle threshold = 30 degrees, step size 1). The reconstructed fiber tracts were parcellated using the AICHA parcellation that had been warped onto each subject's B0 map in subject space. White matter connectivity maps were generated by identifying the number fiber connections passing through both rois and extracting the mean FA from those fibers. Graph analysis using weight conserving measures, i.e., positive strength, was used to describe each subject's white matter connectivity map at the nodal level (wm rois) and to assess group differences (amyloid pos CN vs. amyloid neg CN) and the influence of age and SUVR on white matter connectivity. White matter connectivity is denoted with cFA.

#### Cross-modality analysis

The influence of white and gray matter connectivity on stationary and dynamic functional strength, dispersion was assessed by correlating each nodes strength dispersion with that of its own white (cFA positively correlated with Sdisp) and gray matter (sSneg negatively correlated with Sdisp) connectivity and with the white and gray matter connectivity of every other node. To reflect the temporal aspect of the dynamic analysis, nodal Sdisp was weighted by the time during which this cluster was observed in the individual before correlating it with white/gray matter connectivity measures. To facilitate the interpretation of these cross-modality analyses, the brain was divided into 20 regions (left and right, lateral frontal, medial frontal, cingulate, insula, lateral temporal, medial temporal, lateral parietal, medial parietal, occipital, and subcortical) and significant correlations between rois within a region or with rois in other regions counted (regional cross-modality connectivity matrix). To account for the different number of rois within a region, the connectivity within or between regions was expressed as a ratio (count of significant correlations/no of rois in region with fewer rois). The overall connectivity of each region was determined by summing up the region's entries in the cross-modality connectivity matrix along the x and y axis (region connectivity summary). Upper and lower 99% confidence intervals were calculated for each region summary to identify regions with an increased (>99% upper confidence interval) and decreased (<99% lower confidence interval) cross-modality interaction by bootstrapping (10,000 iterations).

### Statistics

Stationary fMRI analysis/white matter and gray matter connectivity analyses: two-tailed Spearman correlation analyses corrected (FDR *p* < 0.05) for multiple comparisons were used to assess each nodes strength with age and SUVR.

- Cross-modality analyses: one-tailed Spearman correlation analyses corrected for multiple comparisons (FDR *p* < 0.05) were used to identify significant correlations between rois.
- Dynamic analysis: Mann Whitney and one tailed Spearman correlation tests were used to test for differences of the occurrences of different clusters between amyloid pos and amyloid neg CN and to investigate the relationship between cluster counts and SUVR, age and cognitive function. JMP 12.1.0 was used for the cluster analysis, the statistical toolbox in Matlab was used for all other statistical analyses.

## Results

### Functional connectivity

#### Stationary analysis

**Figure 2:**
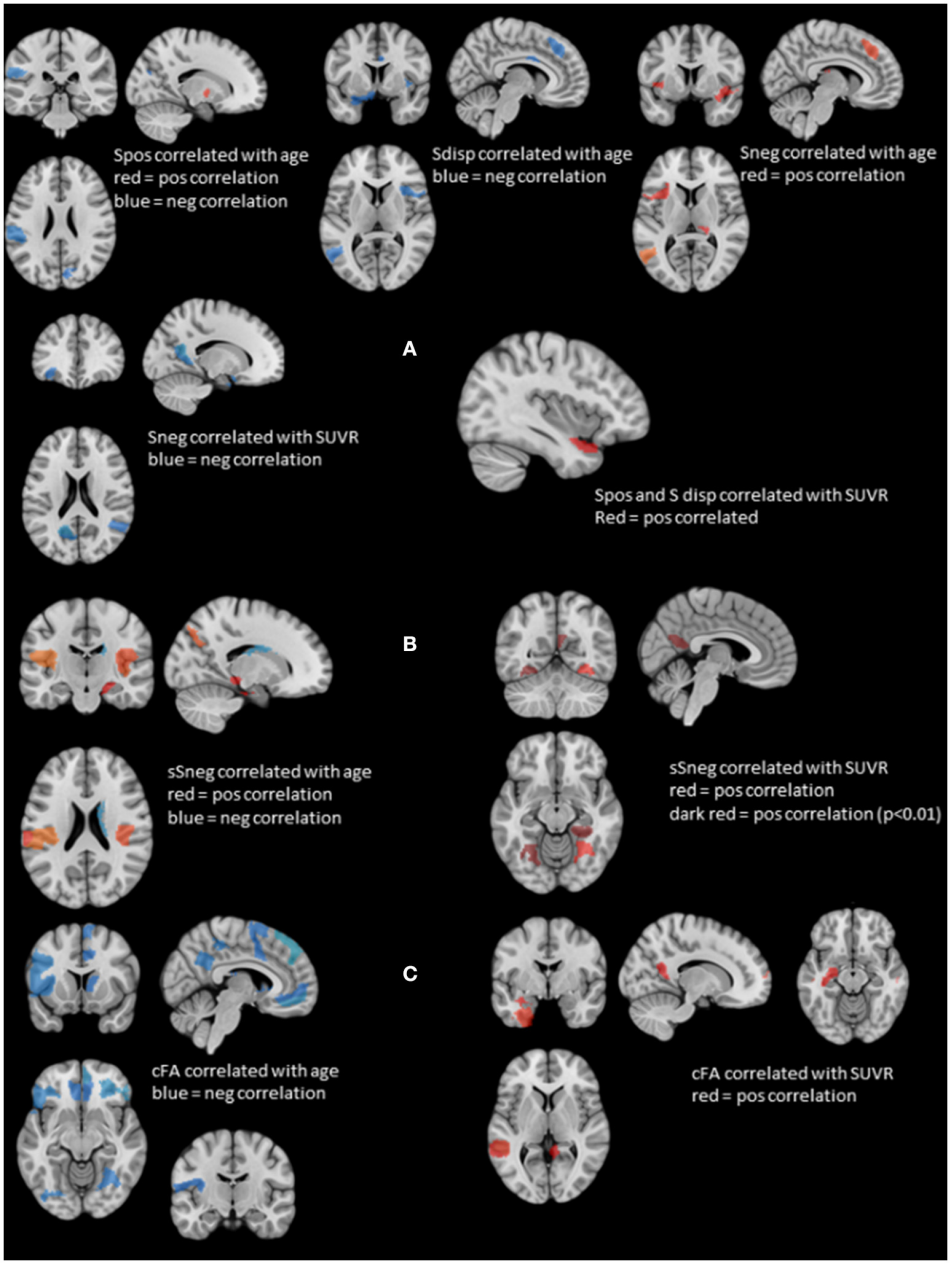
Summary of the structural and stationary connectivity analyses. Regions with positive correlations are displayed in red, those with negative correlations in blue. **(A)** Summarizes the findings of the stationary functional analysis for each of the measures, i.e., positive strength (Spos), negative strength (Sneg) and strength dispersion (Sdisp, calculated as difference between Spos and Sneg) with age and SUVR. **(B)** Displays the findings for atrophy-based gray matter connectivity (please see Methods in text body for details). On the left side regions whose negative structural strength (sSneg) is positively correlated with age, and on the right side regions whose sSneg is positively correlated with SUVR. There was no overlap between regions affected by age and those affected by amyloid load. **(C)** Displays the findings for white matter connectivity (please see Methods in text body for details). cFA is FA weighted stream line count connecting two regions. Correlations with age are displayed on the left and those with SUVR on the right. Again, there was no overlap between regions correlated with age and those correlated with SUVR.

Figure 2A displays rois whose nodal strength correlated with age and SUVR. Age was significantly negatively correlated with Spos of rois in the left supramarginal gyrus and right cuneus and positively with a roi in the right putamen. Sneg was positively correlated with age in rois in the left superior frontal, medial temporal and right thalamus and in the left and right anterior insula and mesial prefrontal region. Sdisp was negatively correlated with age in rois of the left supramarginal gyrus and middle temporal gyrus, right mesial prefrontal gyrus and cingulum and bilateral anterior insula.

Spos and Sdisp were positively correlated with SUVR in the left anterior superior temporal gyrus and Sneg was negatively correlated with SUVR in the left orbital-frontal, anterior insula, parieto-occipital and precuneus region and the right superior temporal region. There were no significant correlations between stationary Spos, Sneg, or Sdisp and short or delayed recall discriminability. Taken together, increasing age induced a shift from positive to negative strength, while SUVR had the opposite effect and demonstrated evidence for a SUVR associated focal hypersynchrony in the left superior temporal gyrus.

#### Dynamic analysis

The number of different clusters or types of activity observed in a subject ranged between 4 and 8 (median: 6). Please see also Table 2. Clusters 1–5 were characterized by a relative decrease of Spos that was accompanied by a decrease of Sneg in clusters 4 and 5 and an increase of Sneg in clusters 1–3. Clusters 6–8 were characterized by an increase of Spos and a decrease of Sneg. Clusters 1–3 and clusters 6–8 represent unbalanced states in which one connectivity type dominates. In the case of clusters 1–3 the balance is shifted toward negative strength indicating the presence of a large number of negative correlations between rois. In clusters 6–8 the balance is shifted toward positive strength consistent with predominately positive correlations between rois. Clusters 4 and 5 represent balanced states during which Spos and Sneg are both slightly reduced. Clusters with similar strength profiles tended to be neighbors indicating that those with less pronounced strength profile represent a transition from one strength profile into another, e.g., from unbalanced negative to balanced. Cluster 8 had the highest Spos and lowest Sneg and consequently the largest Sdisp of all clusters, i.e., represented a highly unbalanced state favoring positive correlations which is consistent with a strength profile of hypersynchrony. Cluster 8 was also the only cluster whose window counts/subject were positively correlated with a subject's SUVR (*r* = 0.42, *p* < 0.03) and negatively with a subject's memory performance (CVLT short free recall, *r* = −0.43, *p* < 0.03, delayed recall *r* = −0.39, *p* < 0.04). Please see Supplementary Table 1 for other clusters.

**Figure 3:**
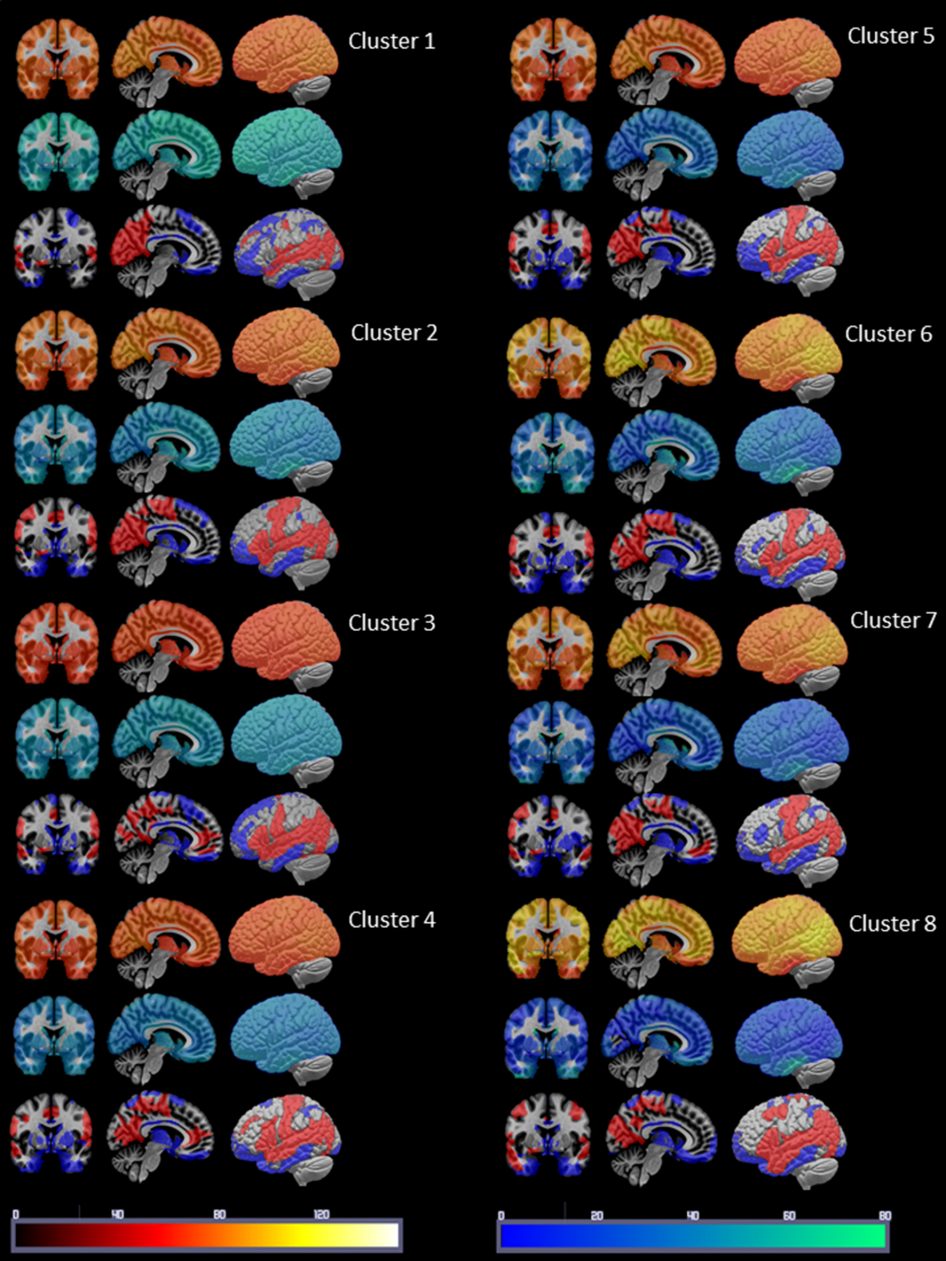
Summary of the cluster characterization. The upper row shows the distribution of Spos in warm colors (please see color bar at the bottom of the figure), the middle row shows the Sneg distribution in cold colors (please see color bar at the bottom) and the lower row the maxima (>75 percentile) of S disp in red and the minima (<25 percentile) in blue. Please see Results section for a description of the findings.

Figure 3 displays the mean nodal Spos (yellow-red) and nodal Sneg (green-blue) and maximal (75–100 percentile, red) and minimal (0–25 percentile, blue) Sdisp nodes for each cluster. Although the global Spos and Sneg of most clusters differed (please see Table 3), the distribution of the respective maxima and minima was quite similar. Although the extent of involvement varied, the mesial temporal region including the inferior temporal and fusiform gyrus and the orbito-frontal region were identified as minimal Sdisp zones in all eight clusters. The minimal Sdisp zones were caused by a lower Spos but higher Sneg compared to other regions. Additional less consistent minimal Sdisp zones were found the dorso-lateral and mesial frontal cortex. The middle and superior temporal gyrus, lateral temporo-parietal region, lateral and medial occipital gyri without occipital poles, and the precuneus were identified as maximal Sdisp zones in all eight clusters. The mesial superior frontal region, anterior cingulate, the paracentral lobules, and pre- and post-central gyri were identified as additional less consistent maximal Sdisp zones. In accordance with its profile in the cluster analysis, cluster 8 was characterized by the highest global mean Spos and lowest global mean Sneg. This shift from Sneg to Spos at the global level was also observed in the lateral temporal lobe where over 50% of its area were identified as maximal Sdisp zones and only 23% as a medium (25–50 percentile) Sdisp zones which was clearly smaller than in other clusters (please see Table 3.). The mesial temporal regions showed the opposite pattern, i.e., only 9% of its area were identified as maximal Sdisp regions but over 55% as medium Sdisp zone. This indicates that large parts of the mesial temporal lobe were not able to engage in the high Sdisp activity observed in the lateral temporal lobes and other extratemporal brain regions. This pattern was only found in Cluster 8.

**Table 3:** Summary of regional strength distribution.

	**Measure**	**Cluster 1**	**Cluster 2**	**Cluster 3**	**Cluster 4**	**Cluster 5**	**Cluster 6**	**Cluster 7**	**Cluster 8**
Global	Spos	76.6 (3.8)	76.7 (1.9)	66.9 (1.5)	70.8 (1.4)	74.3 (1.0)	87.7 (3.1)	81.1 (2.1)	92.9 (3.5)
	Sneg	64.8 (1.4)	57.7 (1.5)	59.1(1.4)	54.8 (0.8)	51.0 (0.6)	52.1 (2.3)	46.9 (1.5)	42.2 (1.8)
	% Low S disp	19.6	20.2	20.1	20.3	20	21.2	21	18.9
	% Medium Sdisp	56.3	50.5	52.1	48.2	51.5	51.2	51.4	51.2
	% High S disp	24.1	29.3	27.8	31.5	28.5	27.6	27.6	29.9
Temporal lateral	Spos	76.5 (5.7)^*^	77.0 (5.4)^*^	67.1 (3.2)^*^	71.2 (4.1)^*^	75.0 (6.1)^*^	86.5 (9.2)^*^	81.0 (4.8)^*^	94.1 (8.5)
	Sneg	62.6 (5.0)^*^	56.8 (5.1)^*^	58.0 (3.1)^*^	53.6 (2.8)^*^	50.0 (4.4)^*^	53.3 (8.3)^*^	46.4 (3.5)^*^	41.6 (4.4)^*^
	% Low S disp	11.7	22.9	19	20.5	21.9	31.4	27.2	26.5
	% Medium Sdisp	55.6	43.5	42.2	35.9	43.4	44.4	41.5	**23.5**
	% High S disp	32.7	33.6	38.8	43.6	34.7	24.2	31.3	**50.0**
Temporal medial	Spos	75.1 (6.6)^*^	74.4 (5.3)^*^	65.2 (2.5)^*^	68.8 (4.2)^*^	71.7 (5.5)^*^	84.2 (11.8)	78.4 (7.1)^*^	86.3 (11.2)
	Sneg	63.5 (5.4)^*^	57.8 (4.8)^*^	58.2 (2.6)^*^	54.4 (3.5)^*^	51.4 (4.7)^*^	54.4 (9.0)^*^	48.2 (5.9)	46.2 (7.6)
	% Low S disp	32.3	41.7	33.2	37.7	42.3	42.6	37.6	35.3
	% Medium Sdisp	46.5	37.1	48.9	49.3	44.5	38.8	43.8	**55.5**
	% High S disp	21.2	21.2	17.9	13	13.2	18.6	18.6	**9.2**

* *Significant different compared to cluster 8, global Spos, all clusters except 1 and 2 different, global Sneg all clusters different. pos, pos strength, Sneg, neg strength, S disp, strength dispersion, low, within 0–25 percentile; high, with 75–100 percentile, % coverage of total lat TL or med TL area. Bold highlights S disp behavior unique to cluster 8*.

The next step was to investigate a potential relationship between memory performance and the observed type of strength re-distribution. To this purpose windows characterized by larger than normal (above upper 99% confidence interval) maximal Sdisp zones and lower than normal (below lower 99% confidence interval) medium Sdisp zones in the lateral temporal regions and windows characterized by smaller than normal (below lower 99% confidence interval) maximal Sdisp and larger than normal (above upper 99% confidence interval) medium Sdisp zones were identified in each cluster and counted for each subject. In cluster 8, the counts of windows with smaller than usual medium Sdisp zones (25–50 percentile) in the lateral temporal lobe were negatively correlated with short free recall (*r* = −0.56, *p* = 0.005) and delayed recall (*r* = −0.53, *p* = 0.008). The counts of windows with larger than usual maximal Sdisp zones in the lateral temporal lobe were also significantly negatively correlated with short recall and delayed recall but these correlations did not survive correction for multiple comparisons. The counts with larger than usual medium (25–50 percentile) Sdisp zones in the mesial temporal region were negatively correlated with delayed recall (*r* = −0.53, *p* = 0.008). The correlations between counts of windows with larger than usual medium Sdisp zones for short recall and those for smaller than usual high Sdisp zones were also significantly negatively correlated with short recall and delayed recall but did not survive correction for multiple comparisons. Taken together, the findings indicate that a shift of the strength distribution toward higher Spos with simultaneous decrease of Sneg in the lateral temporal lobe that excludes a large part of the medial temporal lobe could have an adverse effect on memory performance if they occur frequently or over a longer time. None of the other clusters showed this kind of relationship with memory performance.

### Structural connectivity

Figure 2B shows gm rois whose sSneg was significantly correlated with age. Positive correlations indicating increased connectivity with other gray matter regions with similar degrees of age-related atrophy were found in gm rois in the left supramarginal gyrus, right superior temporal, parahippocampal and parieto-occipital regions and bilateral posterior insula. A roi in the right caudate was negatively correlated with age. A single roi in the right fusiform gyrus was positively correlated with SUVR. When a more liberal threshold was used (*p* < 0.01) additional rois with positive correlations between sSneg and SUVR were found in the left and right fusiform gyrus and the left precueneus. Taken together, age and SUVR were both positively correlated with sSneg. Age-related sSneg increases were diffuse while SUVR-related Sneg increases were restricted to fusiform gyrus and precuneus. There was no overlap between rois showing age-related sSneg increases and rois showing SUVR related sSneg increases.

Wm rois whose cFA was negatively correlated with age were found in left and right orbito-frontal, inferior frontal, occipital, lateral parietal, precuneus and cingulate regions, in left precentral, rolandic, middle frontal, anterior insula and right superior and medial frontal, superior temporal, fusiform and thalamus regions. Wm rois with cFA that was positively correlated with SUVR were found in left superior temporal, hippocampus, fusiform regions and right superior frontal and middle temporal regions and bilateral precuneus (cf. Figure 2C). Taken together, age was negatively correlated with cFA and age-related cFA decreases were widespread but were more common prefrontal. In contrast, cFA was positively correlated with SUVR. Significant correlations were restricted to rois within the temporal lobes and precuneus. There was no overlap between rois correlated with age and those correlated with SUVR.

### Cross-modal correlations

**Figure 4:**
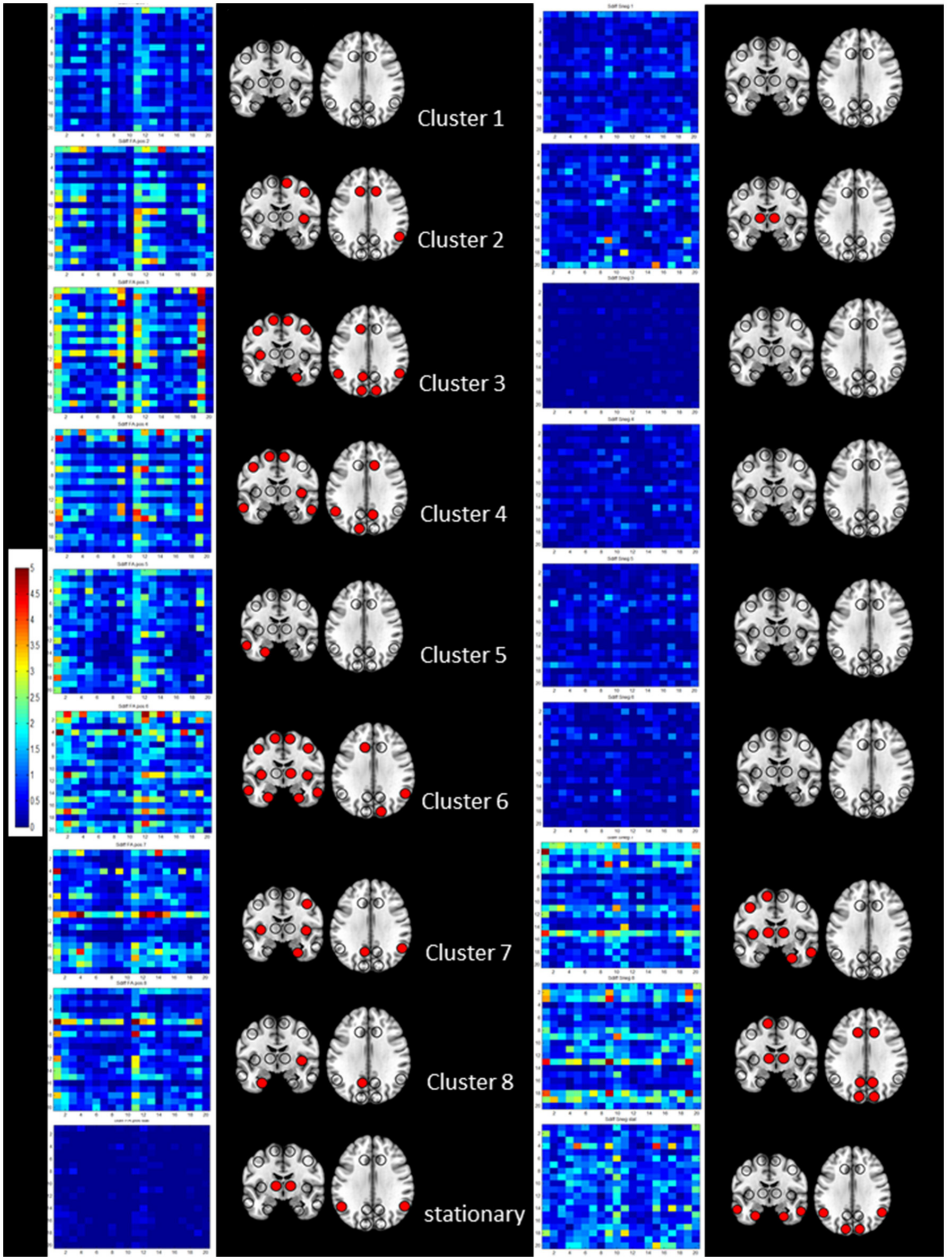
Summary of the results of the cross-modality correlation for each cluster (1–8 from top to bottom) and the stationary analysis (bottom). On the left side significant positive correlations between S disp and cFA are summarized by their regional cross-modality connectivity matrices displaying 20 regions (x from left to right and y top to bottom 1, left lateral frontal, 2, left medial frontal, 3, left cingulate, 4, left insula, 5, left lateral temporal, 6, left medial temporal, 7, left lateral parietal, 8, left medial parietal, 9, left occipital and 10, left subcortical, 11, right lateral frontal, 12, right medial frontal, 13, right cingulate, 14, right insula, 15, right lateral temporal, 16, right medial temporal, 17, right lateral parietal, 18, right medial parietal, 19, right occipital, and 20, right subcortical) and a graphical representation of region connectivity summary where regions with above thresholds are indicated with a filled circle. On the right, significant negative correlations between S disp and sSneg are summarized in the same way.

Figure 4 shows the regional cross-modality connectivity matrices and the graphical representation of the region connectivity summaries for the stationary analyses and each cluster. All clusters except No. 1 had several regions with above threshold positive correlations between Sdisp and cFA indicating that white matter connectivity had a role in maintaining Sdisp regardless of the magnitude of Sdisp. The most prominent Sdisp cFA correlations were found for Cluster 3 and 6 with 13, respectively, 14 above threshold regions. Cluster 8 had only three above Sdisp/cFA threshold regions, one of them the left mesio-temporal region. Cluster 8 and 7, i.e., both clusters with an unbalanced positive strength profile and high Sdisp were the only clusters that had several regions with above threshold negative correlations between Sdisp and sSneg indicating an adverse effect of gray matter atrophy on maintaining activity characterized by high S disp.

## Discussion

There were two major findings: (1) The dynamic functional connectivity analysis revealed paroxysmal phases of unbalanced activity characterized by a widespread increased strength dispersion, i.e., high pos strength associated with low neg strength, consistent with hypersynchrony in cluster 8. The duration of these phases was positively correlated with amyloid load indicating a relationship between amyloid load and the occurrence and severity of these paroxysmal phases. The widespread shift toward positive strength at the expense of negative strength also included large parts of lateral temporal lobes but mostly spared mesial temporal lobe structures, indicating a state of functional disconnection of the mesial temporal region. The duration of this mesial temporal disconnection state was negatively correlated with memory performance. This paroxysmal widespread hypersynchrony seen in the dynamic analysis was only weakly reflected in the traditional time-averaged analysis that showed decreased neg strength in isolated rois in the frontal, parietal and temporal lobes and increased pos strength in the superior temporal lobe. There was no association with memory performance in the time averaged analysis. (2) Amyloid load was associated with an increased white matter connectivity in the left lateral and mesial temporal lobe and precuneus and with an increased atrophy related connectivity in the right fusiform gyrus. The findings of the cross-modality analysis suggest that the increased white matter connectivity enabled the brain to maintain the hypersynchrony and that the altered gray matter connectivity in the mesial temporal lobe contributed to the functional disconnection of this region. Taken together, the findings support the notion that increased amyloid load in CN is associated with phases of widespread paroxysmal hyperconnectivity consistent with hypersynchrony and that these phases could have a negative impact on memory. The findings of the structural analysis suggest that this widespread paroxysmal hypersynchrony depends on an intact structural connectivity, i.e., that they either become less widespread or vanish completely when the neurodegenerative process progresses. The following paragraphs will discuss these findings in more detail and attempt to put them into the context of the current knowledge.

The first major finding of this study was that an increasing amyloid load was associated with increasingly longer paroxysmal states characterized by increased positive strength and simultaneously decreased negative strength resulting in a widespread increased strength dispersion. This hyperconnectivity pattern is consistent with a widespread low level hypersynchrony that has also been described in AD animal models at a very early stage of the disease (Busche and Konnerth, 2016; Shah et al., 2016). Although widespread, the strength dispersion had regional maxima in the medium and superior temporal lobes, lateral temporo-parieto-occipital region and precuneus. Regional strength dispersion minima caused by decreased pos strength with simultaneously increased neg strength were located in in the orbito-frontal region, mesial temporal and inferior temporal region. This strength pattern indicates that the BOLD signal in these regions was anti-correlated to that of the majority of other regions. Interestingly, the other activity states or clusters showed very similar maxima and minima even though the strength dispersion during those phases was far less prominent. This can be interpreted as evidence that paroxysmal hypersynchrony enhances existing strength patterns in the brain rather than re-configuring them. Only about 45% of the CN (14 amyloid neg CN, seven amyloid pos CN) displayed cluster 8 activity in their task-free fMRI. The absence of hyperconnectivity phases in the other amyloid pos CN does not allow for the conclusion that they are free of such episodes though. It is possible that hyperconnectivity phases are less frequent, less severe or less widespread in these subjects and therefore not detected during the 8 min task-free fMRI with the approach used in this study. That being said it is equally possible that the hyperconnectivity phases indeed occur only in a subset of the amyloid pos and neg subjects who share a common unknown predisposition that renders the brain susceptible to paroxysmal hypersynchrony and that increasing brain amyloid enhances that predisposition. It will be necessary to investigate this question in longitudinal studies that acquire task-free fMRI over a longer time, e.g., 20–30 min.

The cognitive performance of participants in this study was within the age appropriate range and not different between subjects with and without increased brain amyloid. Nonetheless memory performance was negatively correlated with the duration of these paroxysmal hypersynchrony phases. This suggests that these phases might negatively affect cognitive function if they become longer or occur more frequently. To better understand how these transient hypersynchrony states interfere with memory performance, their spatial and temporal pattern in the mesial and lateral temporal lobe was investigated in more detail. Compared to other activity clusters, the high strength dispersion zone during cluster 8 activity engaged a larger part of the lateral temporal region (50% compared to 24–44% in other clusters) which led to a smaller moderate strength dispersion zone (23% compared to 35–55% in other clusters). These findings can be interpreted as evidence that the lateral temporal lobe is able to engage in the hypersynchronous activity causing the extreme strength dispersion in other parts of the brain. This was not the case in the mesial temporal region where the high strength dispersion zone was smaller (9% compared to 13–25% in other clusters) but the intermediate strength dispersion zone was larger than that of other activity clusters (55 vs. 31–49%). The increased zone of intermediate strength dispersion indicates that the hypersynchronous activity that dominates other brain regions is not able to completely overcome the anti-correlated BOLD activity in the mesial temporal region resulting in a functional disconnection of these structures during these phases. The finding of a negative correlation between memory performance and the frequency of windows in which extreme manifestations of this mesial temporal disconnection and of the temporal lateral hypersynchronization were observed also supports this hypothesis.

The extreme strength profile of cluster 8 activity, i.e., synchronization of the BOLD fluctuations over a large brain region but failure to engage mesial temporal lobe structures, can be explained by the findings of the structural connectivity analyses. While age had the expected effect on white matter connectivity, i.e., was negatively correlated with white matter connectivity in mostly frontal regions, amyloid load was positively correlated with white matter connectivity in lateral temporal lobe structures and to a lesser degree also in mesial temporal structures and in the precuneus. Although one has to be careful when interpreting DTI findings in regard of white matter integrity/functionality (Jones et al., 2013), the positive correlation with SUVR and the normal cognitive function suggests that amyloid did not have a negative impact on white matter connectivity at this early stage. This is in accordance with other cross-sectional studies that found normal or increased FA in amyloid positive CN in the absence of widespread tau accumulation (Racine et al., 2014; Wolf et al., 2015; Rieckmann et al., 2016; Kantarci et al., 2017). Normal or even increased white matter connectivity facilitates the generation and spread of the hypersynchronous activity hypothesized to be responsible for the prominent strength dispersion characterizing cluster 8 activity. It also supports normal between-region interactions as evidenced in the cross-modality analysis that found one or more regions with above threshold correlations between strength dispersion and white matter connectivity in almost all clusters. In contrast, amyloid had a negative effect on gray matter connectivity in the mesial temporal region as evidenced by the positive correlation between SUVR and atrophy-related connectivity in the right fusiform cortex that was accompanied by more widespread mesial temporal connectivity loss at more liberal statistical thresholds. It seems reasonable to assume that subtle atrophy in the mesial temporal region contributed to the functional disconnection of this region during cluster 8 activity. This assumption is also supported by the findings of the cross-modality analysis. Clusters 8 and 7 that are both characterized by a high strength dispersion show several regions with an above threshold number of negative correlations between atrophy affected gray matter connectivity and strength dispersion.

A SUVR exceeding the threshold used for amyloid positivity in this study indicates a diffuse, widespread pathology with amyloid deposits in the lateral superior temporal, lateral and midline frontal and parietal regions and beyond (Braak and Braak, 1997). The prolonged widespread hypersynchrony phases with maxima in the lateral temporal and temporoparietal, lateral frontal regions, and precueus observed in the amyloid pos CN in this study are consistent with this widespread amyloid pathology. The circumscribed gray matter atrophy and the localized functional disconnection in the mesial temporal region however seem at odds with a diffuse pathology and also with the widely acknowledged observation that amyloid pathology correlates poorly with neurodegeneration. A circumscribed pathology with signs of neurodegeneration is commonly associated with tau pathology. Interestingly, the strength dispersion minima in the mesial, inferior temporal and orbito-frontal regions are not only different from that seem in young controls (cf. Supplementary Figure 1) but also correspond well to the pattern of tau pathology at this stage (Schöll et al., 2016; Pontecorvo et al., 2017; Schultz et al., 2017; Sepulcre et al., 2017). The “dual” association of the mesial temporal structural and functional findings with amyloid (significant correlation with SUVR) on the one hand and with tau (regional preference, neurodegeneration) on the other hand is interesting because it ties into the observation that amyloid facilitates the development of widespread tau pathology that characterizes the clinical manifest stages of AD (Musiek and Holtzman, 2015). The mechanisms of this interaction are still far from clear and are one of the major research topics of the AD field (Pooler et al., 2015; Lewis and Dickson, 2016; Ayers et al., 2017). In the context of this study's findings, it is particularly interesting that neuronal activity is supposed to play a major role in some of the proposed mechanisms. It is tempting to speculate that the type of widespread low level hypersynchronous activity that characterizes cluster 8 could represent a type of neuronal activity that is particularly well-suited to enable tau spreading. If this is true, amyloid pos subjects who show prolonged phases of this activity in task-free fMRI, would be expected to be at higher risk to develop a widespread tau pathology, cognitive impairment and brain atrophy associated with it than amyloid pos subjects who do not show hyperconnectivity phases or only very short phases. Given the negative correlation between atrophy-related gray matter connectivity and functional strength dispersion, it would be expected that the spreading tau leads to the development of widespread gray matter atrophy with secondary impact on white matter connectivity. As a consequence of the increasingly impaired structural connectivity the widespread functional hyperconnectivity of the early stage would be gradually replaced by a widespread hypoconnectivity in the later stages (Schultz et al., 2017). Identifying amyloid pos CN with hypersynchronous phases at an early stage and suppressing that activity with a suitable medication (Bakker et al., 2015) could prevent this hypothesized interaction between amyloid and tau and thus eventually delay or even prevent the development of cognitive impairment.

To our knowledge, this is the first study that combines a dynamic task-free fMRI analysis with white and gray matter connectivity analyses to investigate structure-function associations in amyloid pos and neg CN. There are previous studies that used different approaches of dynamic task-free analysis to investigate functional dynamics in preclinical and early AD. For example, Jones et al. (2012) investigated the dwell time of subnetworks in the dorsal and posterior DMN in AD patients and found shorter dwell times in brain states with posterior DMN contributions and longer dwell times in those with dorsal DMN contributions in AD compared to controls. Demirtaş et al. (2017) used effective connectivity to study global and regional fluctuations of synchronization over the whole range of the AD from healthy controls to fully developed AD and found a monotonous decrease over the disease course. Kang et al. (2017) used regional homogeneity (ReHo) to investigate functional synchronization in amyloid neg and pos CN and found positive correlations between amyloid load and ReHo in the lingual gyrus, left fusiform gyrus, and right middle temporal gyrus in amyloid pos subjects, i.e., evidence for a localized hypersynchrony. Quevenco et al. (2017) finally used a sliding windows approach combined with PCA in CN and found a reduced anterior-posterior connectivity in CN whose cognitive functions worsened over 2 years compared to those who did not decline but no significant association between this connectivity reduction and amyloid load. While these studies clearly show that dynamic task-free fMRI analyses help to better understand the impact of beta amyloid on brain function, the study populations and/or analysis methods differ from the approach used in this study which complicates a comparison of the findings. The same is also true for the only previous study that investigated gray matter connectivity at the single subject level in amyloid pos and neg CN so far. Tijms et al. used a high resolution parcellation to assess similarities of gray matter structure instead of gray matter loss as was done in this study. They used graph analysis designed to look at sparse binary networks to describe gray matter disruptions and found a lower whole brain connectivity density and a less efficient network organization in amyloid pos CN (Tijms et al., 2016).

The study has several limitations. (1) Amyloid load was assessed using a region of interest approach to calculate global SUVR. Given the objective of this study it would have been desirable to use quantitative fluorbetapir maps. However, differences in the acquisition of the PET data at the two sites prevented the reconstruction of quantitative maps. (2) Given the potential association of some of the findings with tau pathology, it would have been desirable to obtain tau PET images as well. However, this was not possible due to budgetary restraints and the limited availability of the tracer at the time of this project. (3) The cross-modality analysis used simple spearman correlations between each and every roi of the two modalities and FDR to correct for multiple comparisons. This approach is not uncommon, but there exist more sophisticated multivariate statistical approaches, e.g., partial least square or sparse canonical correlation, and it cannot be excluded that these would have detected additional interesting associations between modalities. (4) The study population in this study was small and thus these findings have to be considered as preliminary and need to be confirmed in different and ideally larger populations that have amyloid and tau imaging, e.g., the new ADNI.

## Author contributions

SM: Developed hypotheses and analysis methods, performed analysis, manuscript editing and writing; MW: Critical discussion of hypotheses and findings, manuscript editing and writing.

### Conflict of interest statement

MW has been on scientific advisory boards for Pfizer and BOLT Inter-national; has been a consultant for Pfizer Inc., Janssen, KLJ Associates, Easton Associates, in Thought, INC Research, Inc., Alzheimer's Drug Discovery Foundation and Sanofi-Aventis Groupe; has received funding for travel from Pfizer, Novartis, Tohoku University, MCI Group, France, Travel eDreams, Inc., Neuroscience School of Advanced Studies (NSAS), Danone Trading, BV, CTAD ANT Congres; has received honoraria from Pfizer, Tohoku University, and Danone Trading, BV; has research support from Merck and Avid. The other author declares that the research was conducted in the absence of any commercial or financial relationships that could be construed as a potential conflict of interest.

## Supplementary material

Supplementary Figure 1 Summary of cluster characterization in 20 young (mean age: 25.6 ± 4.8, range 21–40) healthy and cognitively normal subjects who were studied with the same magnet and with the same imaging protocol. The task-free data was processed in the same way as described for the older subjects with the exception that the Spos and Sneg z-scores were calculated with all 20 subjects as reference. The cluster analysis identified 8 clusters (4 motion clusters), the remaining clusters are displayed. The upper row shows the distribution of Spos in warm colors (please see color bar at the bottom of the figure), the middle row shows the Sneg distribution in cold colors (please see color bar at the bottom) and the lower row the maxima (>75 percentile) of S disp in red and the minima (<25 percentile) in blue. Please note that the Sdisp minima in the temporal lobes of these young subjects are confined to the parahippocampus and that the maxima extent into the inferior temporal lobe gyri. Finally, even though cluster 4 has a non-balanced positive profile, the dispersion is smaller than that of cluster 8 in the old population.

Supplementary Table 1 Correlations No of cluster counts/subject with amyloid load and memory.
