# Supplementary figures and images for: Amyloid Associated Intermittent Network Disruptions in Cognitively Intact Older Subjects: Structural Connectivity Matters
Source: Front Aging Neurosci. 2017 Dec 19;9:418. doi: 10.3389/fnagi.2017.00418 (PMC5742224; doi:10.3389/fnagi.2017.00418)

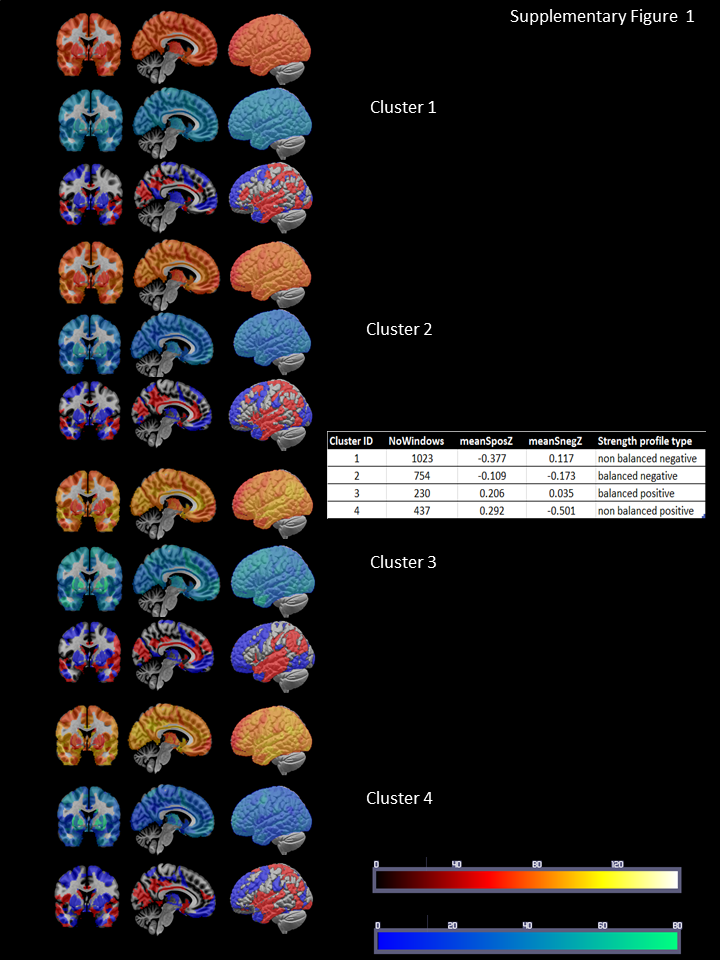

Supplement: Supplementary Figure 1 — Summary of cluster characterization in 20 young (mean age: 25.6 ± 4.8, range 21–40) healthy and cognitively normal subjects who were studied with the same magnet and with the same imaging protocol. The task-free data was processed in the same way as described for the older subjects with the exception that the Spos and Sneg z-scores were calculated with all 20 subjects as reference. The cluster analysis identified 8 clusters (4 motion clusters), the remaining clusters are displayed. The upper row shows the distribution of Spos in warm colors (please see color bar at the bottom of the figure), the middle row shows the Sneg distribution in cold colors (please see color bar at the bottom) and the lower row the maxima (>75 percentile) of S disp in red and the minima (<25 percentile) in blue. Please note that the Sdisp minima in the temporal lobes of these young subjects are confined to the parahippocampus and that the maxima extent into the inferior temporal lobe gyri. Finally, even though cluster 4 has a non-balanced positive profile, the dispersion is smaller than that of cluster 8 in the old population. [file Image1.TIF]
