# Supplementary material for: Amyloid Associated Intermittent Network Disruptions in Cognitively Intact Older Subjects: Structural Connectivity Matters
Source: Front Aging Neurosci. 2017 Dec 19;9:418. doi: 10.3389/fnagi.2017.00418 (PMC5742224; doi:10.3389/fnagi.2017.00418)
Supplement: Supplementary Table 1 — Correlations No of cluster counts/subject with amyloid load and memory. [file Table1.pdf]

Supplementary Table 1. Correlations No of cluster counts/subject with amyloid load and memory

**Cluster**	**SUVR** r (p)	**short free recall** r (p)	**delayed recall** r (p)
1	-0.132 (0.25)	0.048 (0.41)	0.018 (0.46)
2	-0.163 (0.14)	-0.005 (0.38)	-0.102 (0.26)
3	-0.060 (0.26)	-0.040 (0.40)	-0.008 (0.48)
4	0.067 (0.40)	0.190 (0.11)	0.178 (0.12)
5	0.109 (0.24)	-0.063 (0.34)	-0.005 (0.49)
6	-0.001 (0.50)	-0.157 (0.19)	-0.113 (0.26)
7	-0.064 (0.36)	-0.087 (0.31)	-0.080 (0.32)
8	0.420 (0.03)	-0.431 (0.03)	-0.400 (0.04)
